# Combination of ferroptosis and pyroptosis to construct a prognostic classifier and predict immune landscape, chemotherapeutic efficacy and immunosuppressive molecules in hepatocellular carcinoma

**DOI:** 10.1186/s12885-022-09301-0

**Published:** 2022-03-02

**Authors:** Lijun Xu, Qing Zheng, Wenwen Liu

**Affiliations:** 1 Key Laboratory of Gastroenterology and Hepatology, Inflammatory Bowel Disease Research Center, Division of Gastroenterology and Hepatology, Ministry of Health, Shanghai Institute of Digestive Disease, Renji Hospital, School of Medicine, Shanghai Jiao Tong University, Shanghai, 200127 P.R. China; 2 Department of Geratology, Renji Hospital, Shanghai Jiaotong University, School of Medicine, Shanghai, 200127 China

**Keywords:** Hepatocellular carcinoma, Ferroptosis, Pyroptosis, Overall survival, Immune profiles, Immunosuppressive molecules

## Abstract

**Background:**

The induction of ferroptosis and pyroptosis has been highlighted as a novel approach to decide cancer cell fate. However, few studies have systematically explored the role of combining these two novel cell death modalities in hepatocellular carcinoma  (HCC).

**Methods:**

Ferroptosis-related genes (FRGs) and pyroptosis-related genes (PRGs) were retrieved and downloaded from FerrDb and GeneCards database, respectively. A prognostic classifier integrating with prognostic differentially expressed FRGs and PRGs was constructed by the least absolute shrinkage and selection operator (LASSO) algorithm in the TCGA-LIHC dataset and verified using the ICGC (LIRI-JP) dataset.

**Results:**

A total of 194 differentially expressed FRGs and PRGs were identified and named as differentially expressed genes (DEGs) and, out of them 79 were found dramatically correlated with prognosis in HCC. Based on 13 key DEGs with prognostic value, a novel expression signature was constructed and used to stratify HCC patients into 2 groups. Kaplan–Meier analysis demonstrated that high-risk patients had a more dismal prognosis. Receiver operating characteristic curve (ROC) and multivariate Cox analysis confirmed its predictive power and independent characteristic. Immune profile analysis demonstrated that high-risk group had prominent upregulation of immunosuppressive cells, including macrophages, Th2_cells and Treg. The correlation analysis between this signature and immunosuppressive molecules, Immunophenoscore (IPS) and chemotherapeutic efficacy demonstrated that low-risk group had a higher  IC50 of cisplatin, mitomycin and doxorubicin and negatively related with CTLA4, HAVCR2, LAG3, PDCD1, TIGIT and ICIs treatment represented by CTLA4-/PD-1-, CTLA4 + /PD-1-, CTLA4-/PD-1 + .

**Conclusions:**

In this research, a novel expression signature was identified based on FRGs and PRGs in HCC, and this signature could be used to predict prognosis and select patients potentially benefiting from immunotherapies and chemotherapy.

## Background

Hepatocellular carcinoma (HCC) is a prevalent malignancy worldwide which is characterized by increasing incidence and unfavorable prognosis [1, 2]. Although early-stage HCC patients could receive liver resection, transplantation and radiofrequency ablation, many patients still suffer from tumor recurrence [3]. As a novel therapeutic approach, immunotherapies based on immune checkpoint inhibitors (ICIs) have benefited HCC patients in many clinical trials [4]. For unresectable HCC patients, the therapeutic efficacy of atezolizumab plus bevacizumab for overall survival (OS) is superior to that of sorafenib [5]. However, some HCC patients who receive ICIs treatment, such as nivolumab and pembrolizumab fail to show significant improvement in OS [6, 7], which might be due to tumors’ innate resistance to apoptosis [8]. Thus, inducing novel modalities of cell death has become a promising target of antitumor therapeutic strategy. Ferroptosis and pyroptosis are such essential biological processes in HCC [9–11].

As an iron-dependent type of regulated cell death, ferroptosis is characterized by accumulation of lipid peroxidation to lethal levels [12]. Currently, genes identified to regulate this novel form of cell death could be classified into 3 categories: drivers of ferroptosis (DOF), suppressors of ferroptosis (SOF) and others, which could either drive or suppress ferroptosis based on the context [13, 14]. Pyroptosis is a lytic form of regulated cell death characterized by release of many proinflammatory mediators. There are 2 major methods by which dead cells could activate pyroptosis: GSDMD-dependent manner regulated by caspase1/4/5/11 and GSDME-dependent manner regulated by caspase 3 [15–19].

Accumulating evidence has identified the induction of ferroptosis and pyroptosis as a novel approach by which CD8 + T cells could inhibit tumor growth. For instance, CD8 + T cells could release IFN-γ to downregulate SLC7A11 expression, resulting in lipid ROS accumulation and tumor cell ferroptosis [20]. The activation of ferroptosis further promotes antitumor immunity. Besides, CD8 + T cells could release GzmA (GSDMB-cleaving enzyme) and GzmB (GSDME-cleaving enzyme) to induce pyroptosis. Induced tumor cell pyroptosis could activate IL-1β, which is derived from macrophages and required for antitumor immunity [8]. The induction of ferroptosis and pyroptosis could enhance anticancer immunity and suppress tumor growth, suggesting a favorable prognosis for HCC patients. However, few studies have systematically discussed the possibility of combining these 2 cell death modalities in HCC.

Thus, our study focuses on the comprehensive analysis of a combined ferroptosis-related genes (FRGs) and pyroptosis related genes (PRGs) for HCC with regard to prognosis, clinicopathological feature, chemotherapeutic efficacy, tumor-infiltrating immune cells and immunosuppressive molecules.

## Materials and methods

### Acquisition of data, FRGs and PRGs

Gene expression profiling and survival data of 365 HCC patients were obtained from The Cancer Genome Atlas liver hepatocellular carcinoma (TCGA-LIHC) dataset [21, 22]. The scale method provided by R “limma” package was used to normalize gene expression values. Another 231 HCC patients with valid RNA-seq data and survival data from the ICGC (LIRI-JP) dataset were downloaded (Table 1). Gene expression values after read count normalization were used.

**Table 1:** Baseline characteristics of HCC patients involved in this research

Characteristics	TCGA-LIHC dataset (*N* = 365)	ICGC-LINC-JP dataset (*N* = 231)
**Age**		
≤ 60	173 (47.4)	49 (21.2%)
> 60	192 (52.6)	182 (78.8%)
**Gender**		
Male	246 (67.4%)	170 (73.6%)
Female	119 (32.6)	61 (26.4%)
**Grade**		
G1-2	230 (63.0%)	NA
G3-4	130 (35.6%)	NA
Unknown	5 (1.4%)	NA
**T stage**		
T1-2	271 (74.2%)	NA
T3-4	91 (24.9%)	NA
Unknown	3 (0.8%)	NA
**N stage**		
N0	248 (67.9%)	NA
N1	4 (1.1%)	NA
Unknown	113 (31.0%)	NA
**M stage**		
M0	263 (72.1%)	NA
M1	3 (0.8%)	NA
Unknown	99 (27.1%)	NA
**Stage**		
Stage I-II	254 (69.6%)	141 (61.0%)
Stage III-IV	87 (23.8%)	90 (39.0%)
Unknown	24 (6.6%)	0 (0%)
**Child_Pugh class**		
A	216 (59.2%)	NA
B	21 (5.8%)	NA
C	1 (0.2%)	NA
Unknown	127 (34.8%)	NA
**Cirrhosis**		
No	132 (36.2%)	NA
Yes	77 (21.1)	NA
Unknown	156 (42.7%)	NA
**Survival status**		
Alive	235 (64.4%)	189 (81.8%)
Deceased	130 (35.6%)	42 (18.2%)

*HCC* hepatocellular carcinoma, *TCGA* The Cancer Genome Atlas, *LIHC* liver hepatocellular carcinoma, *ICGC* International Cancer Genome Consortium

Then, 173 FRGs and 120 PRGs were retrieved from the FerrDb and GeneCards website, respectively.

### Generation of differentially expressed genes (DEGs) with prognostic value

DEGs between HCC samples and normal ones were identified by R “limma” package in the TCGA dataset and false discovery rate (FDR) < 0.05 was set as the threshold. Then, DEGs were subjected to univariate Cox analysis to screen out FRGs and PRGs with prognostic value and *P* value < 0.05 was regarded as statistical difference. Venn diagram was plotted in which the interaction between DEGs and prognosis-related genes was displayed. Correlation analysis among these prognosis-related DEGs was conducted and an interaction network was analyzed in the STRING database to identify hub genes [23].

### Construction of a combined ferroptosis and pyroptosis signature and assessment of its clinical utility

The least absolute shrinkage and selection operator (LASSO) algorithm with tenfold cross-validation, which could minimize the risk of overfitting was used to shrink and select variables [24]. Some genes with a regression coefficient of non-zero were identified as the optimal predictors for OS and incorporated into this novel signature, whose risk score was calculated according to the normalized gene expression value and its corresponding regression coefficient. The median risk score was then used as the cutoff value to divide HCC patients into 2 groups. The difference of clinicopathological features between high- and low-risk group was analyzed by Wilcoxon signed-rank test. The relationship between clinicopathological parameters and risk score was investigated by Chi-square test.

### Estimation of chemotherapeutic efficacy, ICIs-related molecules and Immunophenoscore (IPS) with this signature

To assess whether this signature was associated with the half inhibitory concentration IC50 of common antitumor drugs and chemotherapeutic efficacy, we applied “pRRophetic” package in R. By constructing the ridge regression model based on Genomics of Drug Sensitivity in Cancer (GDSC) (www.cancerrxgene.org/) cell line expression spectrum and TCGA gene expression profiles, the package could apply pRRophetic algorithm to predict drug IC50. Wilcoxon signed-rank test was implemented to compare the difference of IC50 between different risk groups. To investigate the relationship between this combined ferroptosis and pyroptosis signature and immunosuppressive molecules, we explored the difference of CTLA4, HAVCR2, LAG3, PDCD1 and TIGIT expression between high- and low-risk group using R “limma” package and applied “ggpubr” package to transform the results into a visual violin plot. As a superior biomarker to predict response of anti-PD-1 and CTLA-4 therapies, IPS could calculate the determinants of tumor immunogenicity and depict the cancer antigenomes and intra-tumoral immune profiles. This scoring scheme derived from a panel of immune-related genes, which belong to four classes: suppressor cells, effector cells, immunomodulators or checkpoints, and MHC-related molecules. By averaging the samplewise Z scores of the four classes within the respective category, the sum of the weighted averaged Z score was calculated as the IPS.

### Validation of this combined ferroptosis and pyroptosis related signature

The Kaplan–Meier analysis was conducted to analyze the difference of OS between risk groups. R software was used to visualize the distribution of risk score and survival outcome of each HCC patient. R “timeROC” package was used to calculate area under the curve (AUC) of 1-, 2-, 3-year receiver operating characteristic curve (ROC) to evaluate the predictive ability of this novel signature. Principal component analysis (PCA) and t-SNE analysis were conducted to explore whether this signature could differentiate HCC patients between different risk groups. Uni- and multi variate Cox analyses were conducted to confirm whether this signature could serve as an independent predictor for HCC prognosis.

### Functional enrichment analysis and immune profile analysis

DEGs between different risk groups were screened out by R “limma” package and we set FDR < 0.05 and |log2 fold change > 1| as the threshold. Gene ontology (GO) and Kyoto Encyclopedia of Genes and Genomes (KEGG) analysis [25–27] were then performed to understand the biological function and pathways of these DEGs by using “clusterProfiler” R package. To explore the immune infiltration profiles between different risk groups, we conducted single-sample gene set enrichment analysis (ssGSEA) to calculate the score of 16 immune-cell features and 13 immune-function characteristics [28].

## Results

### Identification of prognostic DEGs

**Fig. 1:**
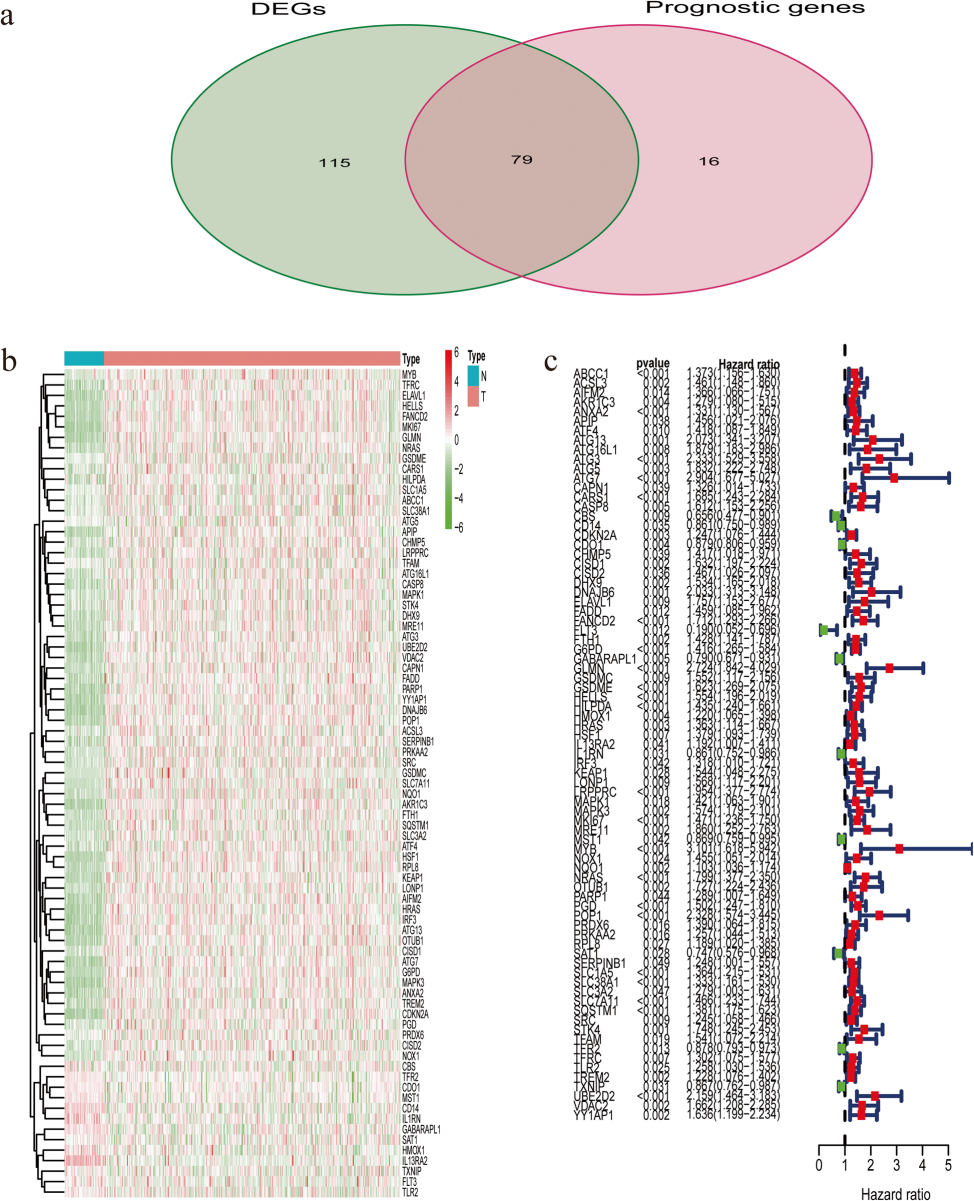
Identification of DEGs with prognostic value in the TCGA-LIHC dataset a. The Venn diagram presented DEGs associated with OS in the univariate Cox regression analysis. b. The heatmap showing 79 prognostic DEGs c. The forest plot displayed the relationship between 79 prognostic DEGs and OS in the univariate Cox regression analysis

**Fig. 2:**
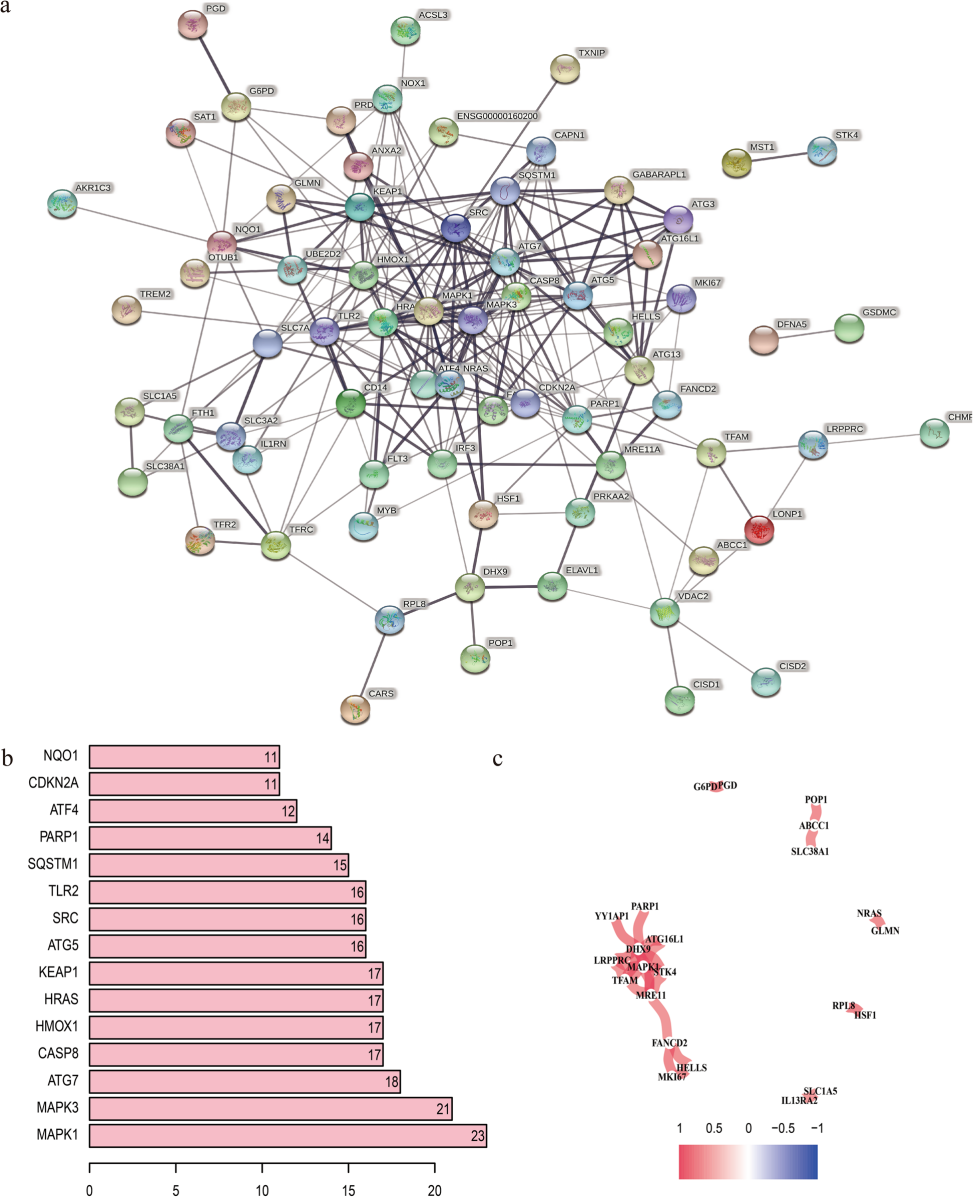
PPI network and correlation analysis of 79 prognostic DEGs. **a** The PPI network among candidate genes obtained from the STRING database. **b** Hub genes with the top 15 degree of interaction. **c** The correlation analysis of candidate genes

A total of 194 DEGs between 374 HCC samples and 50 normal ones were screened out and out of them 79 were identified associated with OS in the univariate Cox analysis (Fig. 1). The protein–protein interaction (PPI) network among these prognostic DEGs were presented in Fig. 2a, in which there were 79 nodes and 230 edges. Genes with the top 15 degree of interaction were identified as hub genes (Fig. 2b). The correlation among 79 prognostic DEGs was displayed in Fig. 2c.

### Construction of a combined ferroptosis and pyroptosis signature

Based on the expression profiles of 79 prognostic DEGs mentioned above, we conducted LASSO regression analysis to develop a novel signature, in which a total of 13 genes were identified as the optimal variables. Among them ATG3, FTL3, G6PD, HILPDA, NRAS, PRDX6, SLC1A5, SLC7A11 were FRGs, SQSTM1 participated in both ferroptosis and pyroptosis, and the remaining 4 genes (GLMN, LRPPRC, MKI67, UBE2D2) were PRGs. The risk score for this novel signature was: [ATG3 expression * (0.0599818988151381)] + [FLT3 expression * (-0.321132320389413)] + [G6PD expression * (0.0881814324303116)] + [GLMN expression * (0.130781902193193)] + [HILPDA expression * (0.119282064768739)] + [LRPPRC expression * (0.00792886569542188)] + [MKI67 expression * (0.0165502840606549)] + [NRAS expression * (0.0916391974243284)] + [PRDX6 expression * (0.114398632925529)] + [SLC1A5 expression * (0.0521211560497305)] + [SLC7A11 expression * (0.0616722848553423)] + [SQSTM1 expression * (0.00940765304518399)] + [UBE2D2 expression * (0.04686256927426)]. The median risk score was then used as the cutoff value to stratify HCC samples into 2 groups, in which there were 182 high- and 183 low-risk cases in the TCGA dataset.

### Evaluation of the clinical utility of this novel signature

**Fig. 3:**
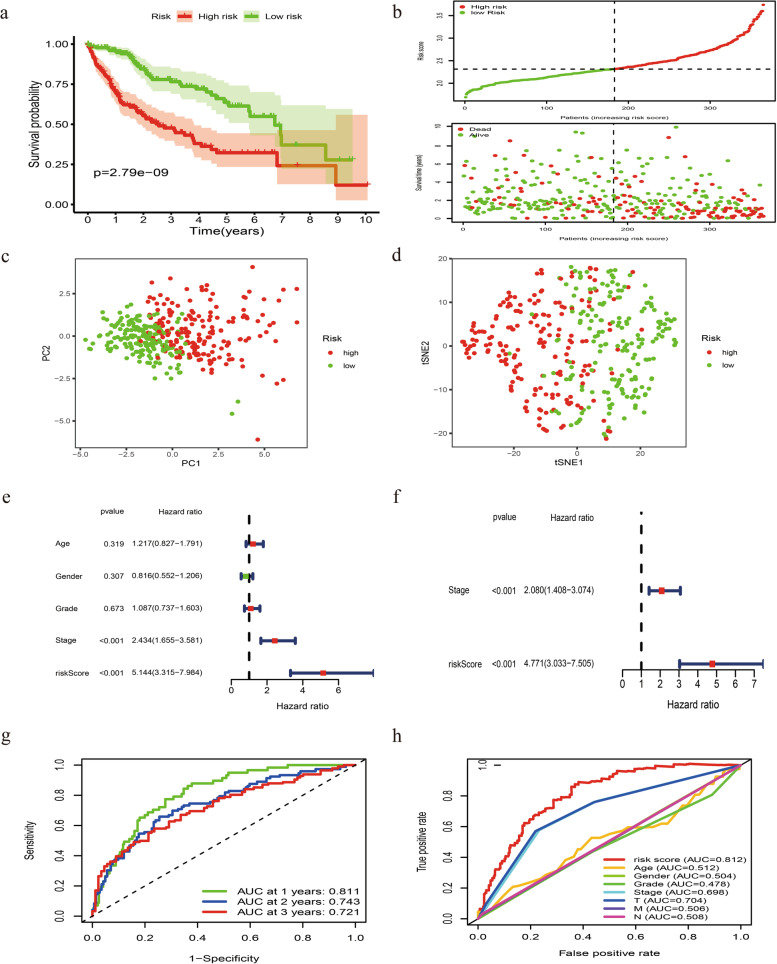
Construction of a combined ferroptosis and pyroptosis signature in the TCGA-LIHC dataset. **a** The Kaplan–Meier curve survival analysis. **b** The risk score curve plot and scatter plot of high- and low- risk HCC patients. **c** PCA plot of the TCGA dataset. **d** t-SNE analysis of the TCGA dataset. **e** Univariate Cox analysis of OS in the TCGA dataset. **f** Multivariate Cox analysis of OS in the TCGA dataset. **g** AUC of 1, 2, 3-year ROC used to assess the predictive ability of this signature. **h** AUC of this signature and clinicopathological parameters

**Fig. 4:**
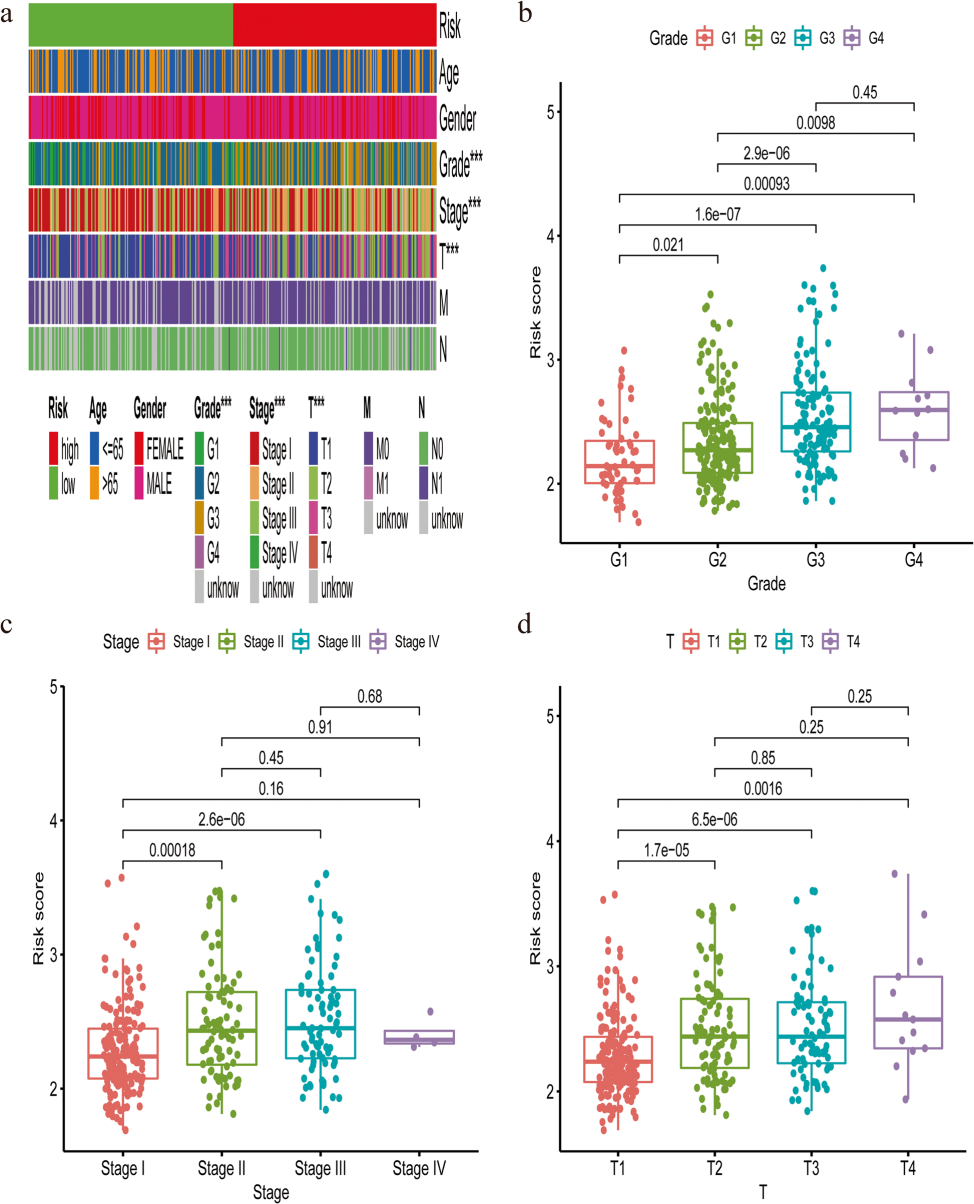
The relationship between clinicopathological features of this novel signature Clinical characteristics (**a**), including tumor grade (**b**), clinical stage (**c**) and T stage (**d**) were significantly associated with the risk and risk score

Kaplan–Meier analysis indicated that high-risk group had a more dismal OS (Fig. 3a, *P* < 0.001). Consistently, as was shown in Fig. 3b, low-risk patients had a lower probability to suffer from earlier death compared with those high-risk counterparts. PCA and t-SNE analysis demonstrated that HCC samples in different risk groups were easily distinguished (Fig. 3c and d). Uni- and multi- variate Cox analyses confirmed that this signature could predict prognosis independent of clinicopathological parameters (Fig. 3e and f). The AUC of 1-, 2-, 3-year ROC for this signature was 0.811, 0.743 and 0.721 (Fig. 3g) and the AUC of this signature was higher than that of clinicopathological indicators (Fig. 3h). Besides, Wilcoxon signed rank test and Chi-square test showed that clinicopathological features (Fig. 4a), including tumor grade (Fig. 4b), clinical stage (Fig. 4c) and T stage (Fig. 4d) were different between risk groups.

### Exploration of the relationship between this signature and chemotherapeutic efficacy, immunosuppressive molecules and IPS

**Fig. 5:**
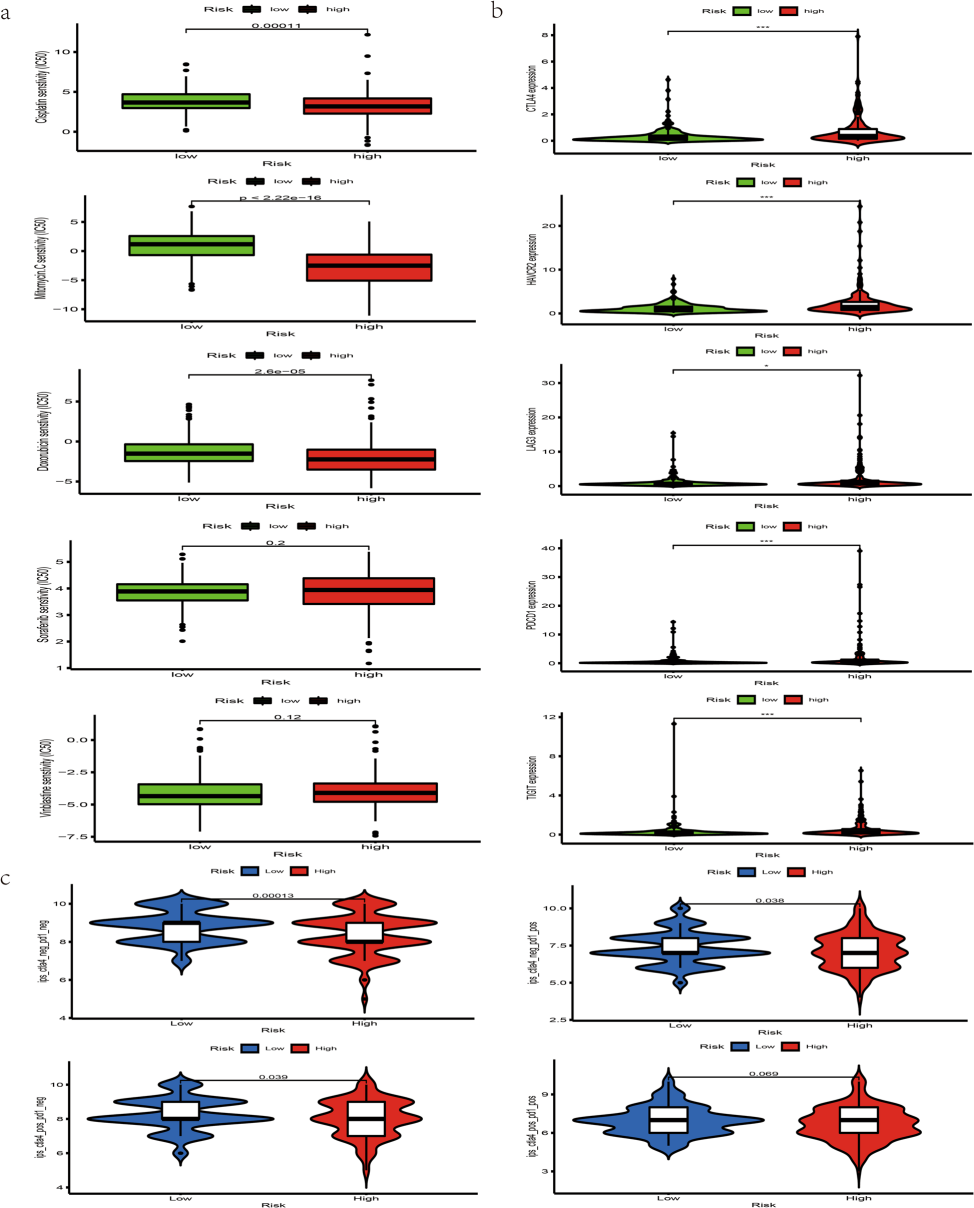
The relationship between this signature and chemotherapeutic efficacy and ICIs-related molecules. **a** Low-risk group had a higher IC50 of cisplatin, doxorubicin and mitomycin. **b** High-risk group was positively related with CTLA4, HAVCR2, LAG3, PDCD1 and TIGIT. **c** Low-risk group was negatively related with ICIs treatment represented by CTLA4-/PD-1-, CTLA4 + /PD-1-, CTLA4-/PD-1 +

By evaluating the role of this signature in predicting the efficacy of common chemotherapeutics, immunosuppressive molecules and IPS, we discovered that low-risk group had a higher IC_50_ of cisplatin, mitomycin and doxorubicin (Fig. 5a) and was negatively related with CTLA4, HAVCR2, LAG3, PDCD1, TIGIT (Fig. 5b) and ICIs treatment represented by CTLA4-/PD-1-, CTLA4 + /PD-1-, CTLA4-/PD-1 + (Fig. 5c).

### Verification of this combined ferroptosis and pyroptosis signature and construction of a nomogram

**Fig. 6:**
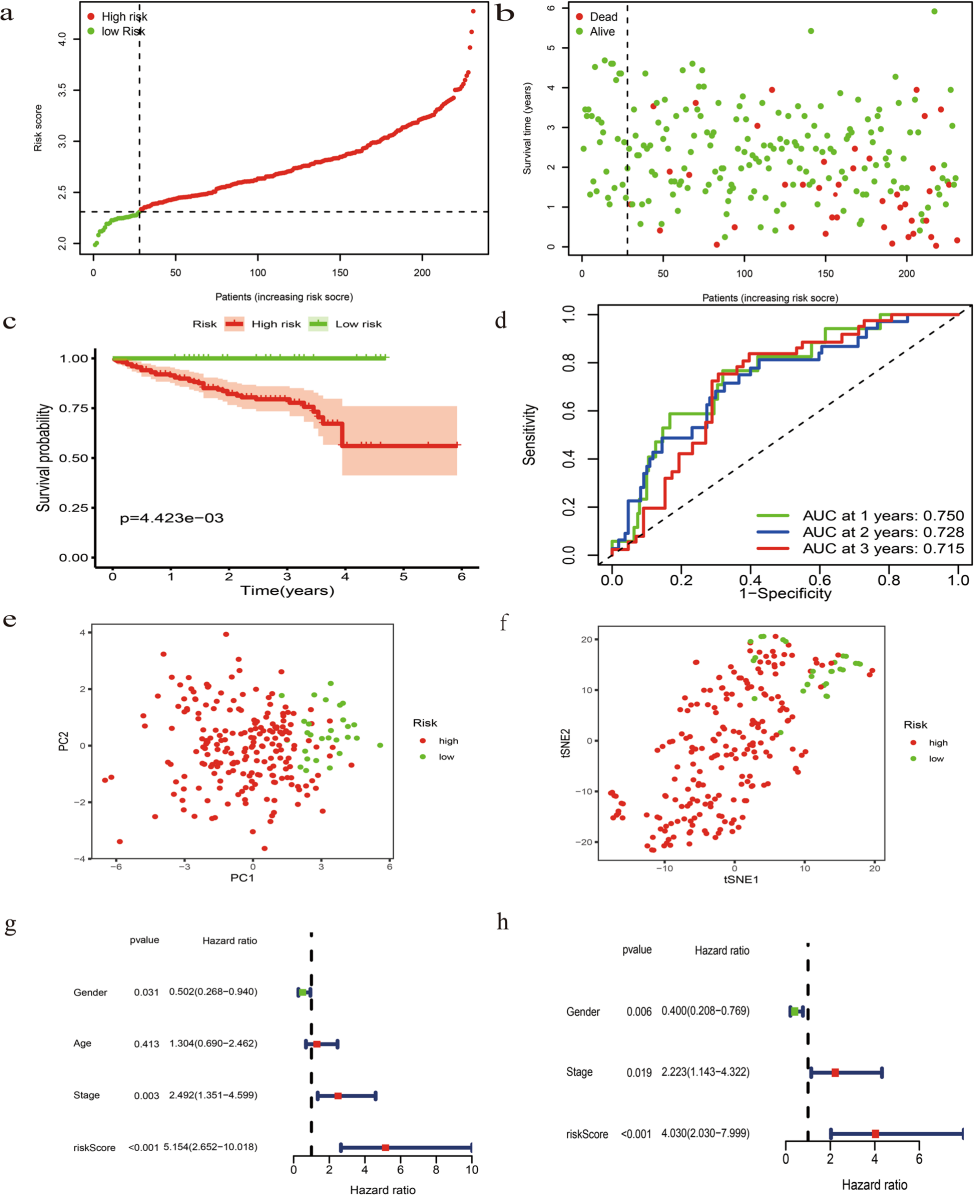
Validation of this novel signature in the ICGC (LIRI-JP) dataset. **a** The risk score curve plot in the ICGC dataset. **b** The risk score scatter plot of high- and low- risk HCC patients. **c** The Kaplan–Meier curve survival analysis. **d** AUC of 1-, 2-, 3-year ROC used to assess performance of this signature in predictive ability in the ICGC dataset. **e** PCA plot of the ICGC dataset. **f** t-SNE analysis of the ICGC dataset. **g** Univariate Cox analysis of OS in the ICGC dataset. **h** Multivariate Cox analysis of OS in the ICGC dataset

**Fig. 7:**
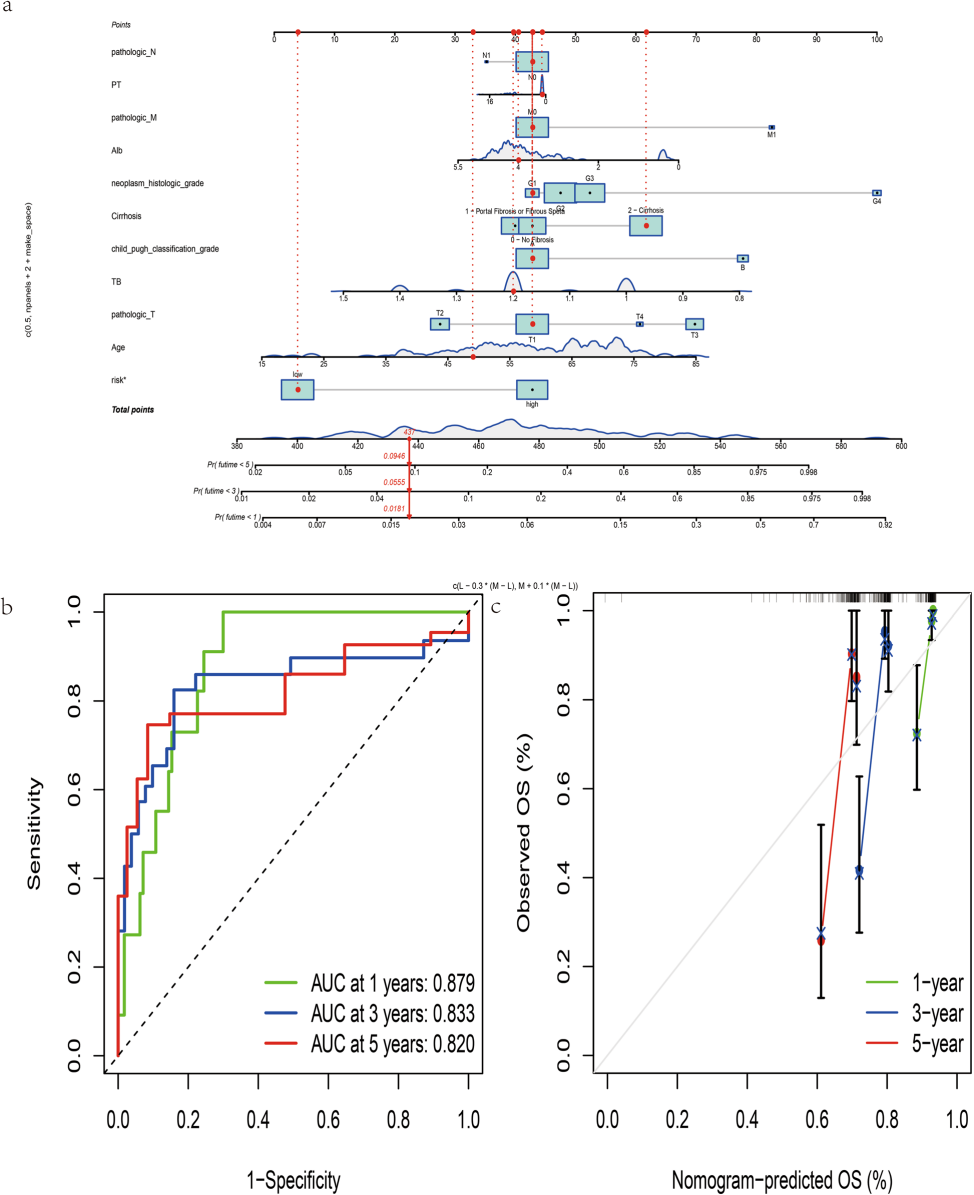
Construction of a nomogram for predicting survival outcome. **a** Nomogram integrating of this novel ferroptosis and pyroptosis-related gene signature and clinicopathological features. **b** AUC of time-dependent ROC curves for nomogram. **c** Calibration plot of nomogram

To assess the predictive ability of this novel signature, ICGC dataset was used as external validation. By calculating the risk score of each HCC sample based on the same formula derived from TCGA dataset, we stratified them into high- or low- risk group according to the cutoff median value (Fig. 6a). Patients with low risk were less susceptible to earlier death and had favorable OS by comparison with those high-risk counterparts (Fig. 6b and c). The AUC of 1-, 2-, 3-year ROC for this signature in the ICGC dataset was 0.750, 0.728 and 0.715 (Fig. 6d). PCA and t-SNE analysis demonstrated that high- and low-risk HCC samples were scattered in two directions (Fig. 6e and f). Uni- and multi-variate Cox analysis confirmed that this signature could predict OS independent of clinicopathological parameters (Fig. 6g and h). To provide a quantitative approach by which clinicians could predict probability of survival in HCC patients, we developed a nomogram by integrating of this novel prognostic signature and clinicopathological characteristics, including age, albumin, total bilirubin, prothrombin time, Child_Pugh classification, histologic grade, TNM stage and liver cirrhosis (Fig. 7a). The predictive accuracy of this nomogram was validated by its discrimination and calibration performance (Fig. 7b and c).

### Functional analysis and immune cell infiltration

**Fig. 8:**
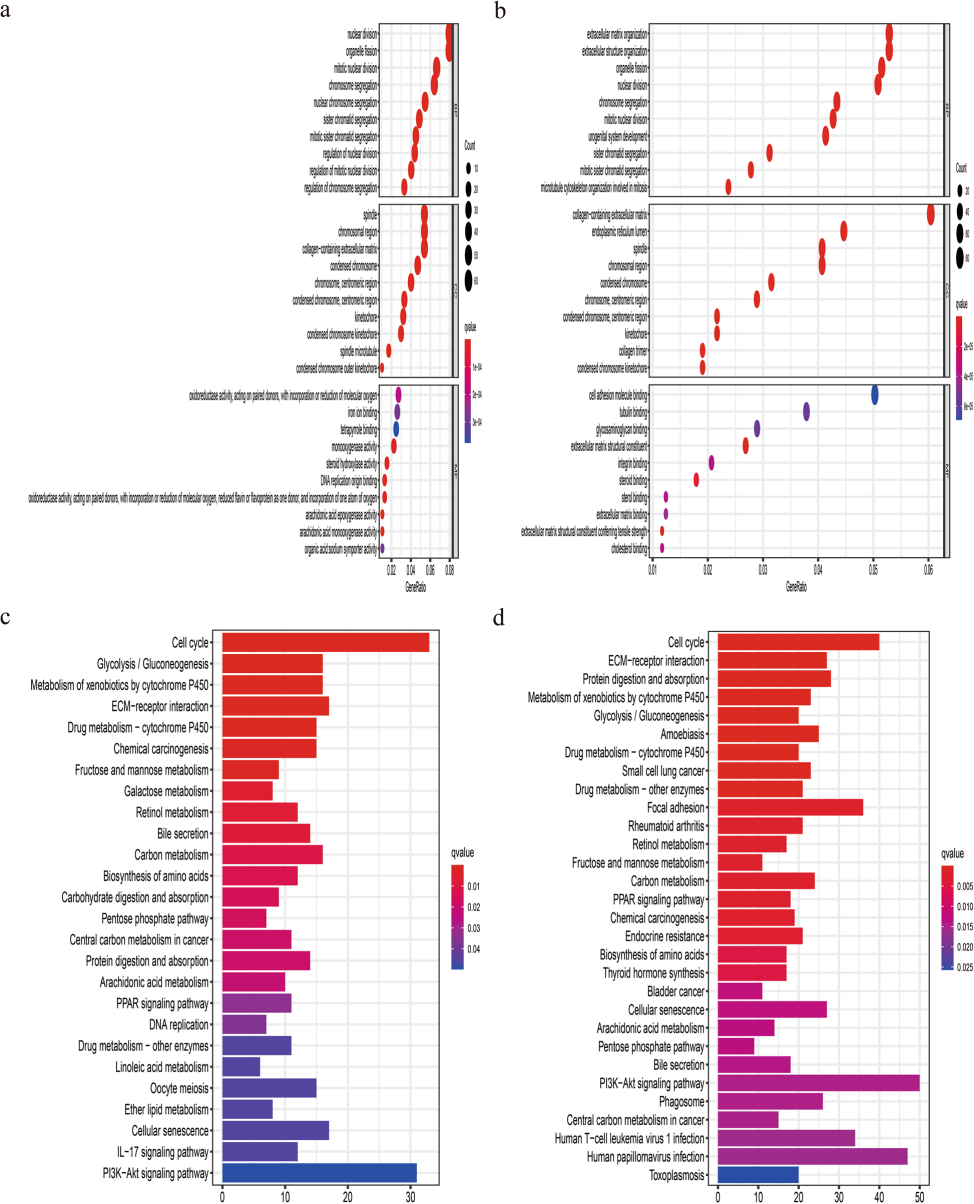
Representative results of GO and KEGG analyses. The most significant GO enrichment and KEGG pathways in the TCGA dataset (**a**, **c**) and ICGC dataset (**b**, **d**) were displayed

To explore the biological function and pathways associated with this signature, we performed GO and KEGG analysis based on DEGs between high- and low-risk group. In both TCGA and ICGC dataset, GO terms for biological process were organelle fission, nuclear division and sister chromatid segregation; for cellular component were mainly associated with chromosome, such as spindle, condensed chromosome, centromeric region (Fig. 8a and b). Besides, the KEGG results revealed that these DEGs mainly participated in cell cycle, PI3K-Akt signaling pathway and cellular senescence (Fig. 8c and d).

**Fig. 9:**
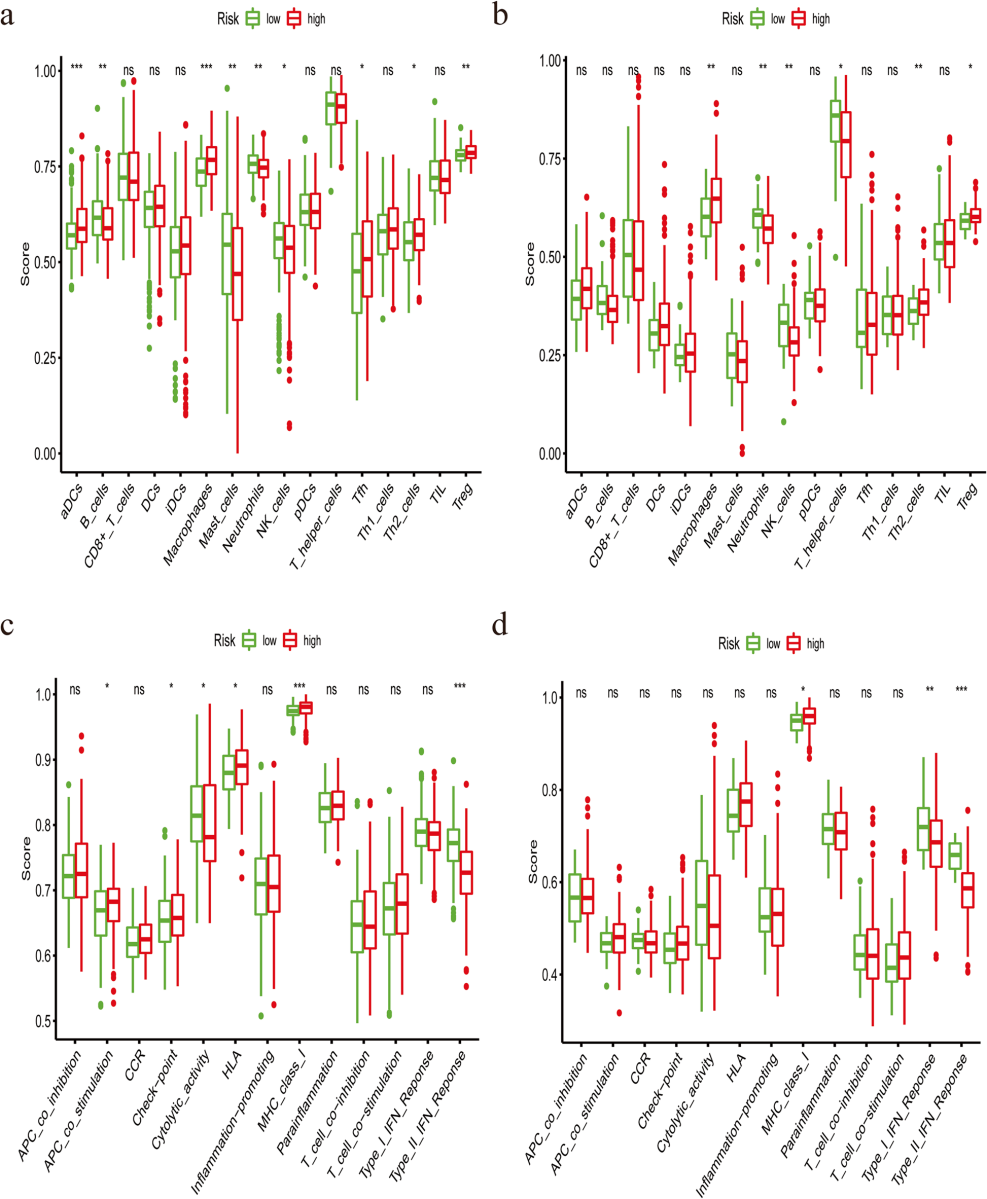
Comparison of the ssGSEA score between different risk groups in the TCGA dataset and ICGC dataset. The score of 16 immune cells (**a**, **b**) and 13 immune-related functions and pathways (**c**, **d**) were displayed in boxplots. Adjusted *P* values were showed as: ns, not significant; *, *P* < 0.05; **, *P* < 0.01; ***, *P* < 0.001

To further investigate the relationship between this signature and immune profiles, we performed ssGSEA to calculate the score of immune-cell features and immune-function characteristics. The score of macrophages, Th2_cells and Treg was higher in high-risk group, while neutrophils and NK cells earned a higher score in low-risk group (Fig. 9a and b). Besides, MHC_class_I and Type_II_IFN_Response was mainly enriched in high- and low-risk group, respectively (Fig. 9c and d).

## Discussion

As two novel cell death modalities, mounting evidence has demonstrated that ferroptosis and pyroptosis are intimately related with tumor progression [29–31]. However, few studies have thoroughly explored the role of combing these 2 novel cell death modalities in HCC. In the present research, we systematically investigated the expression and prognostic significance of 173 FRGs and 120 PRGs in HCC. To our surprise, most of the FRGs (75.2%) and PRGs (59.2%) were found differentially expressed between HCC samples and normal ones, and approximately 40% of them were associated with survival in the univariate Cox analysis. These findings significantly demonstrated the potential role of ferroptosis and pyroptosis in HCC and the possibility of constructing a prognostic expression signature from the perspective of these 2 cell death modalities.

A total of 13 genes were finally incorporated into this novel signature. ATG3, a major autophagy regulator, could promote ferroptosis via degradation of ferritin and accumulation of iron [32]. FLT3 inhibitor could prevent lipid peroxidation to protect cells against oxidative glutamate toxicity, which involves 3 cell death modalities: apoptosis, ferroptosis and necroptosis [33]. G6PD has been considered as a critical pacesetter of pentose phosphate pathway that prevents erastin-induced ferroptosis when it was knocked down [12]. HIF-2a-HILPDA could selectively enrich polyunsaturated fatty acids and acts as a central driver of a ferroptotic cell death [34]. In HL-60/NRAS^Q61L^ cells, HMGB1 knockdown could decrease erastin-induced ferroptosis via RAS-JNK/p38 signaling [35]. PRDX6, an essential family member of nonselenium peroxidases, could protect cells against ferroptotic process and serve as a potential target to enhance antitumor activity of ferroptosis-based chemotherapeutics [36]. SLC1A5, which mediates uptake of neutral amino acids, has been reported to cause ferroptotic cell death when it is suppressed by miR-137 [37]. SLC7A11 is a mechanistic determinant which promotes tumoral lipid oxidation and ferroptosis when it is synergistically suppressed by immunotherapy and radiotherapy [10]. SQSTM1 is passively released in the context of GSDMD-mediated pyroptosis and found to mediate various biological processes, especially autophagy and ferroptosis [38, 39]. Downregulation of GLMN levels could activate inflammasome and pyroptotic cell death of macrophages [40]. Tetherin could prevent T cell pyroptosis by interacting with LRPPRC and preventing the formation of LRPPRC-Bcl-2-Beclin 1 ternary complex [41]. MKI67, a well-known marker of proliferation, was found involved in caspase-3-mediated apoptosis and caspase-1-mediated pyroptosis of CD4 T cells in HIV [42]. Knockdown of UBE2D2, a member of ubiquitin-conjugating enzymes, could significantly reduce SQSTM1 recruitment, which is passively released in the context of GSDMD-mediated pyroptosis [43].

Recent studies have confirmed that ferroptosis and pyroptosis are significantly associated with antitumor activity. Tumor cells undergoing ferroptosis and pyroptosis could recruit tumor-suppressed immune cells and enhance antitumor immunity. To investigate the immune-cell characteristics and immune-function features of this signature, we performed ssGSEA based on DEGs between different risk groups. The results showed that high-risk group of both datasets earned higher scores of macrophages, Th2_cells and Treg, which are thought to promote tumor growth and invasion and tightly associated with dismal prognosis [44–47]. MHC_class_I, a major antigen presenting process, was also enriched in high-risk group. It is possibly because the pore formation with the pyroptotic cellular plasma membrane could release immune stimulants, which could attract activated dendritic cells and thus, promote anti-tumor T cell activity [48]. Besides, cells undergoing ferroptosis could attract dendritic cells by releasing lipid mediators [49]. The scores of NK_cells and Type_II_IFN_Response were higher in low-risk group by the fact that type II IFN is mainly released by NK cells [50], which are major components of immune defense against tumorigenesis [51].

Several shortcomings should be acknowledged in our study. First, this combined ferroptosis and pyroptosis signature was constructed and verified using public dataset. Real-world dataset is required to assess its accuracy and efficacy. Second, the molecular mechanisms between genes identified by this signature and tumor immunity in HCC are not elucidated. Future experimental studies are needed to address this problem.

## Conclusions

In conclusion, a combined ferroptosis and pyroptosis signature was constructed for HCC, which was tightly associated with prognosis, clinicopathological features, immune profiles, chemotherapeutic efficacy and immunosuppressive molecules. The molecular mechanisms between this signature and tumor immunity are largely unknown and require further experimental investigation.

## Data Availability

The datasets generated and/or analysed during the current study are available in the TCGA-LIHC project (https://portal.gdc.cancer.gov/repository) and ICGC database (https://dcc.icgc.org/).

## Abbreviations

HCC: Hepatocellular carcinoma
ICIs: Immune checkpoint inhibitors
OS: Overall survival
DOF: Drivers of ferroptosis
SOF: Suppressors of ferroptosis
FRGs: Ferroptosis-related genes
PRGs: Pyroptosis-related genes
DEGs: Differentially expressed genes
FDR: False discovery rate
LASSO: The least absolute shrinkage and selection operator
IC_50_: The half inhibitory centration
AUC: Area under the curve
ROC: Receiver operating characteristic curve
PCA: Principal component analysis
GO: Gene ontology
KEGG: Kyoto Encyclopedia of Genes and Genomes
ssGSEA: Single-sample gene set enrichment analysis
